# Association exceptionnelle d'un syndrome de Fenton et d'une fracture ipsilatérale de la tête radiale: à propos d'un cas et revue de littérature

**DOI:** 10.11604/pamj.2015.20.373.6063

**Published:** 2015-04-15

**Authors:** Amine Belmoubarik, Marouane Abouchane, Mohamed Amine Mahraoui, Yassir Elandaloussi, Ahmed Reda Haddoun, Mostafa Frikh

**Affiliations:** 1 Centre Hospitalier Universitaire Ibn Rochd, Casablanca, Maroc; 2 Centre Hospitalier Marc Jacquet Melun, France

**Keywords:** Syndrome de Fenton, Fracture du scaphoïde carpien, capitatum, tête radiale, Fenton syndrome, carpal scaphoid fracture, capitatum, radial head

## Abstract

Les auteurs rapportent une observation rare d'un jeune homme de 31 ans qui a présenté une association lésionnelle faite de syndrome de Fenton (fracture scapho capitale) avec fracture de la tête radiale homolatérale survenue suite à une chute aux escaliers. Il s'agit d'une observation décrivant une association lésionnelle exceptionnelle; Traité chirurgicalement en urgence par voie postérieure dorsale au niveau du poignet avec arthroplastie de la tête radiale. L’évolution radio clinique était satisfaisante. Le mécanisme du syndrome de fenton demeure l'objet de controverses. L'association lésionnelle à la fracture de la tête radiale est exceptionnellement décrite dans la littérature. Nous discuterons les mécanismes, notre attitude thérapeutique, et l’évolution de cette entité clinique à travers l'analyse de cette observation.

## Introduction

Le syndrome de fenton ou fracture scapho-capitale est une lésion rare (1), survenant lors de traumatisme du poignet de haute énergie. Elle est presque toujours associée à d'autres lésions du carpe (2). Nous décrivons une observation originale d'un syndrome de fenton associé à une fracture de la tête radiale homolatérale. Association lésionnelle exceptionnelle, témoignant d'une atteinte complexe, et soulevant des problèmes d'ordre éthiopathogénique, thérapeutique, fonctionnel et pronostique que nous essayerons d'analyser à travers notre observation, vu l'originalité de cette association.

## Patient et observation

**Figure 1:**
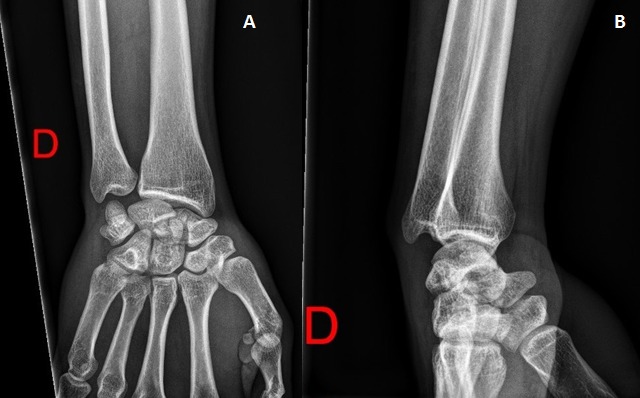
Radiographie du poignet droit de face et de profil: fracture transtuberculaire du scaphoide carpien associée à une fracture du capitatum réalisant le syndrome de Fenton (A,B)

**Figure 2:**
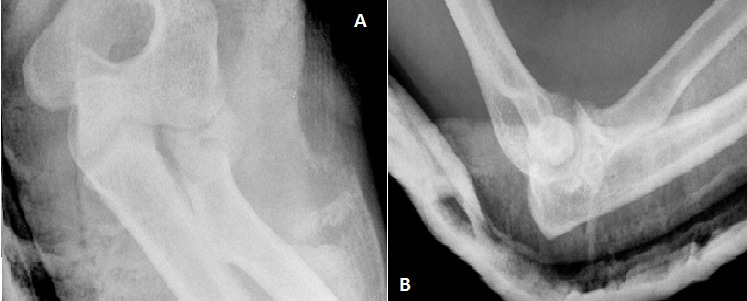
Radiographie du coude droit de face et de profil: facture comminutive de la tête radiale Mason III (A,B)

**Figure 3:**
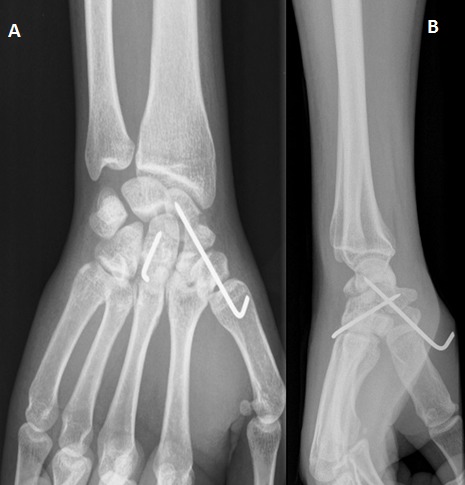
Radiographie du poignet droit de face et de profil: embrochage scapho-capital (A,B)

**Figure 4:**
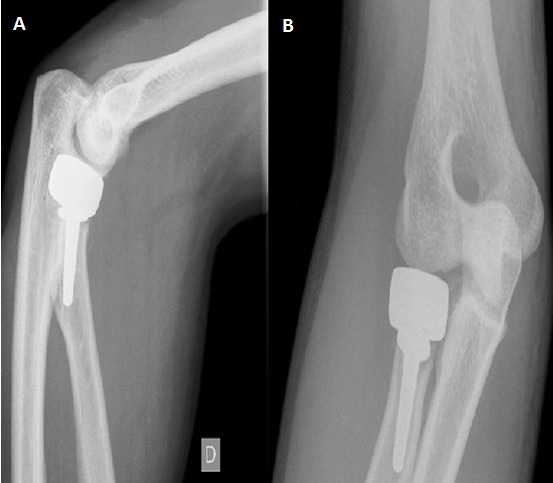
Radiographie du coude droit de face et de profil: arthroplastie de la tête radiale (A,B)

C'est un patient de 31 ans, de sexe masculin, droitier, ouvrier, ramené aux urgences suite à une chute aux escaliers avec réception sur la paume de la main gauche poignet en hyper extension. L'examen clinique trouve une impotence fonctionnelle totale, douleur, ‘dème important du poignet, une douleur au niveau du coude à la mobilisation sans déficits vasculo-nerveux associés. La radiographie du poignet en incidence de face et de profil et des incidences du scaphoïde montre une fracture transtuberculaire du scaphoïde, associée à une fracture complexe du pôle proximal du capitatum avec une rotation de 180° (Figure 1), réalisant un syndrome de Fenton de type I selon Vance et Al (8). La radiographie du coude objective une fracture comminutive de la tête radiale Mason III (Figure 2). Le patient a été opéré, par voie d'abord postérieure dorsale du poignet. Devant la comminution du pole proximal du capitatum et du scaphoïde, nous avons réalisé dans l'immédiat un embrochage scaphoidien avec réduction capitale et ostéosynthèse par embrochage capital (Figure 3). Pour la fracture de la tête radiale a été indiquée une arthroplastie (Figure 4). Le membre supérieur droit a été immobilisé dans une manchette plâtrée prenant le pouce pendant soixante quinze jours. La rééducation commencée après ablation du plâtre et des broches, a permis la récupération d'un poignet stable, mobile, indolore avec légère diminution de la force par rapport au poignet gauche. Le patient été satisfait au recul de neuf mois. On n'a déploré aucune limitation fonctionnelle au niveau du coude dont la rééducation était immédiatement démarrée au décours de l'arthroplastie.

## Discussion

Le syndrome de Fenton, lésion rare [1], peut être isolé mais le plus souvent associé à une luxation péri lunaire ou fracture de voisinage [2, 3]. Classiquement le traitement consiste en une reposition du pôle capital proximal et embrochage. Dans notre cas, elle associée à la fracture du scaphoïde et de la tête radiale homolatérale. Aucun cas similaire, n'ayant été décrit auparavant dans la littérature [4]. L’éthiopathogénie n'est pas unanime concernant le mécanisme lésionnel scaphocapital; chaque auteur avance une hypothèse physiopathologique différente, notre cas semble s'approcher du mécanisme lésionnel tel que décrit par Stein et Johnson [5]. Concernant l'association lésionnelle (notre observation), nous pensons que la chute sur le poignet en extension entraîne par la même occasion la fracture du scaphoïde qui permet dans un premier temps la poursuite du mouvement d'extension faisant ainsi rapprocher la marginale radiale postérieure du radius du capitatum et entraînant à ce moment là, la fracture capitale [6]; Notre hypothèse éthiopathogénique est que l’énergie résiduelle du traumatisme est transmise en miroir cette fois-ci en amont faisant ainsi piéger la tête radiale entre l'axe diaphysaire radial et le capitellum qui se trouve fracturée par mécanisme de casse-noix. La fracture du capitatum passe souvent inaperçue, la recherche de lésions associées à la fracture du scaphoïde doit être toujours de mise, en l'occurrence à distance du point d'impact. Rand [6, 7] insiste sur l'importance de la tomodensitométrie du poignet en urgence qui trouve tout son intérêt dans la recherche de lésions associées et l’établissement d'un bilan lésionnel correct et précis. L'attitude thérapeutique étant par principe chirurgicale comme le préconise Wesely [7, 8], visant la réduction du poignet et la stabilisation des fractures par vis ou broches, cette chirurgie doit répondre aux impératifs de conservation de l'anatomie vasculaire des os du carpe, minimisant ainsi les risques de pseudarthrose ou nécrose céphalique. Notre abord était dorsal, suivi par capsulotomie postérieure, réduction première du capitatum et stabilisation du scaphoïde et du capitatum par deux broches. Notre choix d'arthroplastie de la tête radiale type Guepar était justifié par la comminution importante et par l'association lésionnelle carpienne, nous avons pensé soustraire le patient au risque d'un éventuel syndrome d'Essex Lopressti, de conflit ulnocarpien ultérieur et de raideur associée, en vue d'une rééducation précoce du coude [9]. A l'ablation des broches, sous anesthésie locale, à trois mois, après consolidation des foyers de fracture scaphoidien et capital, l'examen clinique révèle des mobilités du poignet: extension à 30°, flexion à 10°, inclinaison radiale à 5°, inclinaison cubitale à 5°; pronosupination à 0-130°. La reprise du travail a eu lieu au 6éme mois après le traumatisme. Au recul de neuf mois: le poignet est indolore, la force est légèrement diminuée par rapport à la main gauche, les mobilités du poignet sont: extension à 70°; flexion à 50°, inclinaison radiale à 10°, inclinaison cubitale à 10°; pronosupination à 0-180°. Le malade a été satisfait du résultat.

## Conclusion

Le syndrome de Fenton est une lésion rare, à rechercher devant toute luxation périlunaire du carpe. L'association à une fracture de la tête radiale étant exceptionnelle. Le traitement ne peut être que chirurgical faisant appel à l'ostéosynthèse des lésions du carpe et de la tête radiale permettant une récupération fonctionnelle rapide et satisfaisante.
